# Supplementary material for: Learning to live with interfering neighbours: the influence of time of learning and level of encoding on word learning
Source: R Soc Open Sci. 2019 Apr 10;6(4):181842. doi: 10.1098/rsos.181842 (PMC6502395; doi:10.1098/rsos.181842)
Supplement: Experimental Stimuli [file rsos181842supp1.docx]

| **List 1** |  |  |  | **List 2** |  |  |
| --- | --- | --- | --- | --- | --- | --- |
| **Word** | **Novel word** | **Type** |  | **Word** | **Novel word** | **Type** |
| torso | tarso | Natural |  | chalk | coalk | Natural |
| lettuce | lestuce | Natural |  | sperm | scerm | Natural |
| PIGEON | PIGERN | Natural |  | hamster | hemster | Natural |
| WALNUT | WALNOT | Natural |  | GALAXY | GANAXY | Natural |
| GARLIC | GARNIC | Natural |  | MEADOW | MEAROW | Natural |
| POTATO | PORATO | Natural |  | KIDNEY | KIDLEY | Natural |
| embryo | elbryo | Natural |  | BAMBOO | BALBOO | Natural |
| lagoon | lafoon | Natural |  | fungus | fulgus | Natural |
| artery | artely | Natural |  | helium | hesium | Natural |
| raisin | raibin | Natural |  | turnip | turwip | Natural |
| album | alfum | MANMADE |  | robot | rogot | MANMADE |
| ladle | lamle | MANMADE |  | attic | atric | MANMADE |
| vodka | vopka | MANMADE |  | HELMET | HOLMET | MANMADE |
| ANCHOR | AMCHOR | MANMADE |  | COFFIN | COLFIN | MANMADE |
| TARMAC | TALMAC | MANMADE |  | PARCEL | PARGEL | MANMADE |
| CRADLE | CRAGLE | MANMADE |  | VIOLIN | VIODIN | MANMADE |
| SLEEVE | SLEERE | MANMADE |  | TATTOO | TARTOO | MANMADE |
| velvet | velcet | MANMADE |  | luggage | lutgage | MANMADE |
| bakery | bamery | MANMADE |  | gadget | gaddet | MANMADE |
| canteen | calteen | MANMADE |  | picnic | picsic | MANMADE |
|  |  |  |  |  |  |  |
| **List 3** |  |  |  | **List 4** |  |  |
| **Word** | **Novel word** | **Type** |  | **Word** | **Novel word** | **Type** |
| ozone | odone | Natural |  | lemon | leton | Natural |
| ulcer | ulper | Natural |  | tornado | ternado | Natural |
| SPIDER | SPIMER | Natural |  | BANANA | BANARA | Natural |
| BEETLE | BRETLE | Natural |  | CELERY | CEDERY | Natural |
| TENDON | TANDON | Natural |  | OYSTER | OGSTER | Natural |
| TOMATO | TORATO | Natural |  | FOSSIL | FOSTIL | Natural |
| mussel | mulsel | Natural |  | mammal | mamkal | Natural |
| pollen | polfen | Natural |  | almond | alsond | Natural |
| armpit | arfpit | Natural |  | peanut | peanit | Natural |
| baboon | batoon | Natural |  | EYELID | EYENID | NATURAL |
| siren | sisen | MANMADE |  | foyer | fojer | MANMADE |
| chimney | chimtey | MANMADE |  | abbey | abdey | MANMADE |
| razor | rafor | MANMADE |  | NAPKIN | NASKIN | MANMADE |
| TEAPOT | TEAWOT | MANMADE |  | FABRIC | FABLIC | MANMADE |
| NEEDLE | NERDLE | MANMADE |  | MOSAIC | MODAIC | MANMADE |
| PILLAR | PILTAR | MANMADE |  | PENCIL | PUNCIL | MANMADE |
| GUITAR | GUITUR | MANMADE |  | caravan | cagavan | MANMADE |
| laundry | launtry | MANMADE |  | ghetto | ghetlo | MANMADE |
| pulpit | pulrit | MANMADE |  | saucer | saurer | MANMADE |
| liquor | lituor | MANMADE |  | APRON | ALRON | MANMADE |
